# The influence of advertising on compulsive buying – The role of persuasion knowledge

**DOI:** 10.1556/JBA.2.2013.018

**Published:** 2013-12-06

**Authors:** Kalina Mikołajczak-Degrauwe, Malaika Brengman

**Affiliations:** Department of Business, Faculty of Economic and Social Sciences and Solvay Business School, Vrije Universiteit Brussel, Brussel, Belgium

**Keywords:** compulsive buying, attitudes towards advertising, ad avoidance, persuasion knowledge

## Abstract

*Background and aims:* The growing concern over compulsive buying (CB) among consumers has led to vast amount of research examining the antecedents of this maladaptive behaviour. The focus of previous research was, however, mainly on examining the internal, psychological factors contributing to CB. The current research, on the other hand, sheds light on one of the external triggers which can possibly stimulate CB, namely advertising. *Methods:* An online survey has been conducted to identify the attitudes and scepticism towards advertising as well as ad avoidance and persuasion knowledge among a sample of 582 Belgian consumers. Furthermore, all participants were screened with regard to compulsive buying tendencies. *Results:* This research provides evidence that positive attitudes towards advertising can lead to CB. An important factor in this relation is persuasion knowledge. *Conclusions:* The study results lead to the conclusion that people higher in persuasion knowledge dispose less positive attitudes towards advertising which can subsequently prevent them from engaging in CB. Moreover high scores on scepticism towards advertising and ad avoidance among Belgian consumers in our sample point to a need for advertisers to modify their practices in order to gain more trust from consumers. This study also shows that advertising in particular attracts and seems to affect an already disadvantaged group of people – namely compulsive buyers.

## Introduction

Each year corporations spend large amounts of money to tailor and personalize their marketing messages and to discover new tactics to encourage consumers’ repeated consumption (Workman & Paper, 2010). One way to increase the number of shopping trips to the store is advertising. As a result of enormous expenditures and the attention companies pay to advertise their products and services effectively, consumers are exposed to more persuasive advertising pressure than ever before (Neuner, Raab & Reisch, 2005). Although these advances stand to benefit manufacturers and retailers, the influence of advertising can have a devastating effect on a specific vulnerable group of consumers, namely compulsive buyers.

Compulsive buying (CB) refers to “a consumer’s tendency to be preoccupied with buying that is revealed through repetitive buying and a lack of impulse control” (Ridgway, Kukar-Kinney & Monroe, 2008). It is believed that changes in consumer culture could influence the development and growth of CB among consumers (Neuner et al., 2005). As a result of this shifting consumer culture, buying has become more than a means to satisfy physical needs: it provides pleasure and relaxation (Millan & Howard, 2007) and has become a way of expressing one’s identity (Dittmar, Long & Meek, 2004), gaining social status (Han, Nunes & Drèze, 2010) and even regulating one’s emotions (Dittmar, Long & Bond, 2007; Elliott, 1994). When buying is uncontrolled though, that can lead to serious negative consequences for the person affected as well as for society as a whole (O’Guinn & Faber, 1989). Compulsive buying has often been found to cause extreme levels of debt, anxiety and relationship and family problems (O’Guinn & Faber, 1989; Ridgway et al., 2008). Although a vast amount of research has been conducted with regard to the CB phenomenon, within different fields, such as psychology, psychiatry and consumer behaviour, the focus was mostly on revealing the prevalence, comorbidity, psychological antecedents and socio-demographical correlates of compulsive buying (e.g., Joireman, Kees & Sprott, 2010; Mueller et al., 2010; O’Guinn & Faber, 1989). Only very few studies have examined the influence of marketing mix factors on compulsive buying (e.g., Kukar-Kinney, Ridgway & Monroe, 2012; Vicdan, Chapa & de Los Santos, 2007). Yet, investigating the influence of these factors on CB can provide additional insights into the CB phenomenon and demonstrate that besides being motivated internally, CB can also be triggered by the environmental context in which the consumer finds him/herself. This knowledge can further help policy makers, but also responsible marketers and retailers to adjust their practices in a way that prevents this vulnerable group of consumers to spend more than they want to or can afford.

To this end, the goal of the present study is to examine how attitudes towards advertising, scepticism towards advertising and ad avoidance relate to compulsive buying. Moreover we will investigate whether the relationship between persuasion knowledge (PK) – a person’s confidence in his/her ability to understand marketers’ tactics (Bearden, Hardesty & Rose, 2001) – and CB is mediated by his/her attitude towards advertising. Furthermore, we will compare compulsive and non-compulsive buyers with regard to the degree to which they feel exposed to advertising and which advertising media they feel influenced by most. Finally, the research findings and implications will be discussed.

## Conceptual Framework

### Attitudes towards advertising and compulsive buying

Consumers are exposed to hundreds of commercial messages every day (Arens, Weigold & Arens, 2007). The ultimate goal of advertising for manufacturers and retailers is to seduce consumers and to stimulate them to purchase products. It has, however, always been a challenge to make sure that consumers perceive advertising as something positive. Starting from the 1970s, different study results have consequently concluded that public attitudes towards advertising are rather unfavourable (e.g., Alwitt & Prabhaker, 1992; Mittal, 1994; Zanot, 1981), with the exception of one telephone based survey of over 1000 adult consumers (Shavitt, Lowrey & Haefner, 1998), which revealed public’s positive attitudes towards advertising. On the other hand, research shows that positive thoughts concerning advertising affect the attitudes towards products advertised (e.g., Mackenzie & Lutz, 1989). For example, an expensive and intensive advertising campaign is perceived as a signal of high product quality (Kirmani, 1990). Although advertising does not directly increase the willingness to pay, consumers are more prone to buy advertised products in comparison to products that are not being advertised (Haan & Moraga-González, 2011). This way advertising can increase firm’s profits (Joshi & Hanssens, 2010). Researchers have also demonstrated that there is a positive relationship between ad attitude and brand cognition (Biehal, Stephens & Curlo, 1992; Brown & Stayman, 1992). Moreover, a positive attitude towards a brand significantly impacts the intention to buy that brand (Brown & Stayman, 1992; Homer, 1990). We can therefore conclude that positive attitudes towards advertising influence intentions to buy advertised products.

The relationship between compulsive buying and attitudes toward advertising has been investigated by Kwak, Zinkhan and DeLorme (2002). In their conceptualisation compulsive buying tendencies should create negative attitudes towards advertising. Moreover they posit that this relationship is moderated by exposure to TV commercials and TV shows. The latter appeared to be true in a Korean but not in a U.S. sample.

The results of Kwak et al.’s (2002) study were, however, surprising to us. Based on the results, the authors concluded that CB is negatively related to attitudes towards advertising. In our conceptualization though, we expect this relationship to be positive. According to the social comparison theory (Festinger, 1954), individuals have a basic drive to evaluate their own opinions and abilities through comparison with others. These comparisons might be up- or downward. The upward comparisons with ‘better’ others lead to negative self-evaluations, whereas downward comparisons with ‘worse’ others lead to self-enhancement. Since the content of advertising is mostly filled with idealized images, exposure to such ads can result in a negative comparison and an increased need to acquire the advertised material goods (Lee, Lennon & Rudd, 2000; O’Guinn & Faber, 1989). In the same way that positive attitudes towards advertisements strengthen the desire to posses advertised goods (Brown & Stayman, 1992), we believe that these positive attitudes can increase compulsive buying. The reason is that compulsive buyers are characterized by low self-esteem (d’Astous, 1990; Hanley & Wilhelm, 1992), suffer often from depression (Ergin, 2010; Sneath, Lacey & Kennett-Hensel, 2009) and score high on materialism (Dittmar, 2005; Rose, 2007; Johnson & Attmann, 2009). They might therefore also be more prone to buy advertised products as a result of social comparison (via ads) and negative self-evaluation. Thus we hypothesize that:

**H1:** Attitudes towards advertising are positively correlated with compulsive buying.

### Compulsive buying, scepticism and ad avoidance

Positive attitudes towards advertising can generate profit for companies, if they manage for their brands to obtain a salient and prominent position in the consumer’s mind (Haan & Moraga-González, 2011). However, people do not always perceive advertising in a positive way. In fact, a number of studies indicate that consumers tend to have distrustful attitudes towards advertising (e.g., Alwitt & Prabhaker, 1992; Mittal, 1994; Shavitt et al., 1998). The misleading information often presented in ads is one of the most important reasons of the decrease in consumers’ trust towards ads (Darke & Ritchie, 2007). Prior research regarding that topic has mostly focused on identifying the content of misleading advertising. For example, an incomplete comparison such as: ‘Brand X is faster acting’ suggests that brand X is better than others, but does not explicitly mention the source of comparison (faster than what?) (Shimp, 1978). Another example of deception involves claims of brand superiority over other brands such as: ‘Brand X is better than any other’ (Snyder, 1989). This kind of deceptive advertising claims can lead consumers to *scepticism toward advertising* – ‘the tendency not to believe the information claims in advertisements’ (Obermiller & Spangenberg, 1998). It is a sort of defensive consumer reaction (Darke & Ritchie, 2007) that protects consumers from being misled by advertisements encountered later in the future. Though scepticism towards ads makes advertising less efficient and can limit consumers’ abilities to benefit from honest, attractive offers, it can also prove advantageous to consumers by reducing the risk of being deceived (Darke & Ritchie, 2007). We believe moreover that consumers’ scepticism towards ads will reduce their chance to engage in compulsive buying by simply reducing their motivation to possess advertised products. Hence, we expect that:

**H2:** Scepticism towards advertising is negatively related to compulsive buying.

According to Shavitt et al. (1998), personal attitudes towards ads influence consumers’ exposure and attention to advertising. For example, Speck and Elliott (1997) and Cho (2004) demonstrated that consumers’ negative attitudes towards ads can result in *ad avoidance,* defined as ‘all actions by media users that differentially reduce their exposure to ad content’ (Speck & Elliott, 1997). As ad avoidance reduces the possibility of being confronted with idealized images from ads, which could result in a negative comparison and increased need to acquire the advertised material goods among compulsive buyers (O’Guinn & Faber, 1989), we believe that ad avoidance will reduce the possibility of engaging in compulsive buying. Therefore we posit that:

**H3:** Attitudes towards advertising are negatively correlated with ad avoidance.

**H4:** Ad avoidance is negatively correlated with compulsive buying.

### The role of persuasion knowledge

A major challenge faced by consumers is that of understanding marketers’ actions in order to form valid attitudes about influence agents. According to Friestad and Wright (1994) consumers develop knowledge about persuasion throughout their life. This knowledge is to be used by a consumer to interpret, evaluate and respond to influence attempts from advertisers and salespeople. More specifically *persuasion knowledge* (PK) refers to consumers’ knowledge and beliefs about a number of advertising related issues, including beliefs about, marketers’ persuasion goals, marketers’ tactics, the effectiveness and appropriateness of those tactics, as well as beliefs about one’s own coping goals, one’s own coping tactics (Friestad & Wright, 1994).

Previous research on persuasion knowledge in advertising has focused mainly on identifying persuasive content of advertising messages. For example, Burnkrant and Unnava (1995) found that the use of self-referencing in ads (such as ‘You know that…’) increased message elaboration and persuasion when message arguments were strong. Still, Meyers-Levy and Peracchio (1996) found that while a moderate increase in self-referencing enhances persuasion, an extreme increase actually undermines it. Although advertising messages obviously have a persuasion goal (Haugtvedt & Wegener, 1994; Peracchio & Meyers-Levy, 1994), the consumer’s knowledge about the goals and tactics of persuasion agents’ can influence their attitudes (Friestad & Wright, 1994). For example, individuals high in self-esteem are more difficult to persuade as compared to individuals low in self-esteem (Wood & Stagner, 1994). That is because persons with high self-esteem are more confident in their own judgements and are less concerned with social rejection than people low in self-esteem. For the same reason consumers with high self-esteem are more predisposed to doubt advertising claims, rather than believe whatever is presented (Boush, Friestad & Rose, 1994). We believe therefore that consumers who are low in persuasion knowledge will be less aware of tactics used in advertising. Their attitudes towards advertising will therefore be more positive than among consumers with high PK. Positive attitudes towards advertising will in turn lead to a higher risk of engaging in compulsive buying. Therefore we hypothesize that:

**H5:** The relationship between persuasion knowledge and CB is mediated by attitudes towards advertising.

**Figure 1.**
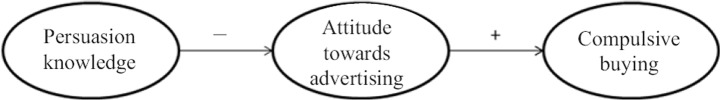
Persuasion knowledge and compulsive buying – the mediating role of attitude towards advertising (hypothesized model)

### The influence of advertising on compulsive buying

Next to identifying the persuasion knowledge of compulsive buyers, their attitudes towards advertising and the extent of scepticism and ad avoidance among them, we wanted to investigate whether compulsive consumers are aware of their exposure to advertising in their daily lives and whether they believe to be influenced by it. While we assume that compulsive buyers are equally aware of their exposure to advertising as non-compulsive buyers, we expect that they will feel more vulnerable to it. As compulsive buyers are aware of their weakness and maladaptive proneness to buy, they will probably also feel more influenced by advertising than non compulsive buyers. Therefore we hypothesize that:

**H6:** Compulsive buyers feel more vulnerable to advertising than non compulsive buyers via (a) TV, (b) magazines, (c) billboards, (d) the Internet.

## Methods

### Measures

*Compulsive buying measure.* The tendency to buy compulsively was measured using the 6-item scale developed by Ridgway et al. (2008). This scale does not include items concerning income and financial consequences, it incorporates both characteristics of obsessive–compulsive behaviour as well as the impulse-control dimensions of buying and is the first to appropriately assess the extent of compulsive buying tendency in the general population of consumers. Four items are measured on seven-point Likert scales from *strongly disagree* to *strongly agree* and two items are measured on seven-point frequency scales from *never* to *very often*. In the current study reliability (Cronbach’s alpha) of this scale was .82 and item-total correlations were all above .47. The average value of the CB index was 15.85, the median value was 15 and the range across respondents was 6–42. According to Curran, West and Finch (1996) rules of thumb for normal distribution (skewness ≤2.0 and kurtosis ≤7.0), the CB measure appeared to be normally distributed with skewness = .834 and kurtosis = 1.013.

*Attitude toward advertising.* From a broad measure of *Consumer sentiment toward marketing,* developed by Gaski and Etzel (1986), measuring a person’s attitude towards marketing practices, one scale specifically, namely *Advertising for products* was used in the current study. This subscale assesses a person’s general attitude towards advertising with 7 items on five-point Likert scales (from *agree strongly* to *disagree strongly*). The reliability (Cronbach’s alpha) of this scale was .75. Two items had item-total correlations below .30. The average value of the *Attitude toward advertising* was 2.39, and the range across respondents was 1–4.71. The measure appeared to be normally distributed with skewness = .242 and kurtosis = –.124. Two items (*Most advertising provides consumers with essential information* and *I enjoy most ads*) were reversed for a convenient interpretation in the structural relationship: higher scores reflect more favourable opinions toward advertising.

*Scepticism toward advertising.* The degree of scepticism a person exhibits towards commercials, particularly concerning the motive of the advertiser, was measured with a five-point Likert-type scale developed by Boush et al. (1994). The measure focused originally on commercials shown on television. We adopted the scale for the current study by omitting the word ‘TV’ in all items, which enabled us to measure a person’s scepticism with commercials in general (not only TV commercials). In the current study reliability (Cronbach’s alpha) for this scale was .78 and item-total correlations were all above .48, except for one item *(Commercials are different from TV programs in the way they try to influence you)* with a correlation of .28. The average value of the scale index was 5.46, and the range across respondents was 2–7. The *Scepticism toward advertising* measure appeared to be normally distributed with skewness = –.303 and kurtosis = .720.

*Ad avoidance.* Six items measuring the frequency (from *never* to *very often*) with which a person avoids ads. The scale is based on Speck and Elliott’s (1997) original measure of *Ad avoidance* (television/magazines), but slightly modified by adding a few items measuring avoidance of ads on the Internet and received via traditional or e-mail. A reliability analysis yielded satisfactory results for the scale with Cronbach’s alpha .77 and item-total correlations all above .36 (see Table 1).

**Table 1.** Item-total correlations for ad avoidance measure

		Item-total correlation
1.	Ignore ads	.557
2.	Switch channels during commercials	.621
3.	Fast forward commercials	.524
4.	Close online pop-up ads without watching them or activate pop-up blocker	.548
5.	Delete promotion/advertisement e-mails without reading them	.618
6.	Don’t allow advertisement mails (post) in my mailbox or throw them away before reading	.363

*Persuasion knowledge.* To measure a person’s confidence in his/her knowledge regarding the tactics used by marketers in their efforts to persuade consumers, we used the *Persuasion knowledge* scale from the broader measure of *Consumer self-confidence* by Bearden et al. (2001). Consumers were asked to rate the degree to which the items are characteristic of them on seven-point Likert scales (from *extremely uncharacteristic* to *extremely characteristic*). The reliability (Cronbach’s alpha) for this 6-item scale was .87. All items had item-total correlations above .55. The average value of the scale was 5.5, and the range across respondents was 1.7–7. The measure appeared to be normally distributed with skewness = –.699 and kurtosis = 1.442.

For an overview of the internal consistency and descriptive statistics of all measures used in the study, see Table 2.

### Sampling and data collection procedure

Data for the current study was collected over a four-month period in the beginning of 2012. Several methods have been used to circulate our online questionnaire. First of all we used invitations on online forums such as *Flair* and *Libelle* (women magazines popular in Belgium), different shopping forums (including a forum for shopping addicts) and others. We have advertised the survey in our university newsletter and asked colleagues and friends to forward the questionnaire. To encourage potential respondents we promised an incentive of one IPod Nano and 10 cinema tickets for 11 randomly chosen respondents.

**Table 2.** Overview of internal consistency and descriptive statistics of measures

		Cronbach’sα	*M*	*SD*	Skewness	Kurtosis
1.	Compulsive buying	.82	15.85	6.090	.834	1.013
2.	Attitude towards advertising	.75	2.39	.660	.242	–.124
3.	Scepticism towards advertising	.78	5.46	.768	–.303	.720
4.	Ad avoidance	.77	5.75	.953	–.983	1.190
5.	Persuasion knowledge	.87	5.50	.789	–.699	1.442

*Note:* Standard error of skewness = .103; standard error of kurtosis = .205.

A total number of 582 Dutch speaking respondents actually participated in the study. After a careful data cleaning procedure (only Belgian adults, aged 17 years or older, were taken into account), 565 participants were retained from the primary dataset, which served as the basis for the further analyses. The sample was clearly dominated by female participants (68.8%). The age range was 17–81 years with a mean of 42 years and a median of 43 years. 73.6% of the respondents was married, cohabiting or in a relationship and 43% did not have children yet. 71.2% had at least a Bachelor diploma and 62.8% was employed. With regard to the net monthly income the most represented group (30%) earned between €1.500 and €2.200, and 17.2% had no income at all.

### Ethics

The study procedures were carried out in accordance with the Declaration of Helsinki. The Institutional Review Board of the Vrije Universiteit Brussel approved the study. All subjects were informed about the study and all provided informed consent.

## Results

The correlation matrix of all the variables, shown in Table 3, provides support for some of our hypotheses. Taking into account these intercorrelations between the independent variables the structural equation modelling has been conducted to compare different models’ fit to the data and strengthen the validity of the results.

**Table 3.** Correlation matrix of measures

		1	2	3	4	5
1.	Compulsive buying	–				
2.	Attitude towards advertising	.139^*^	–			
3.	Scepticism towards advertising	–.068	–.463^*^	–		
4.	Ad avoidance	–.058	–.355^*^	.207^*^	–	
5.	Persuasion knowledge	–.131^*^	–.160^*^	.236^*^	.215^*^	–

*Note:* High scores on attitude towards advertising scale indicate more positive attitudes. High scores on all other scales reflect stronger respective tendencies. ^*^ *p* < .01 (two-tailed).

The processing patterns were subsequently analysed by means of *Partial Least Squares* (PLS) (Ringle, Wende & Will, 2005). The rules of thumb for sample size were met (565 respondents) meaning that the sample size is sufficient to obtain stable estimates (Chin, 1998). The quality criteria are listed in Table 4.

**Table 4.** Overview of the constructs’ quality indicators

	AVE	Composite reliability	*R*^2^
Attitudes towards advertising	.530	.849	.017
Compulsive buying	.546	.877	.039
Scepticism towards advertising	.569	.867	.224
Ad avoidance	.550	.829	.087
Persuasion knowledge	.596	.898	

The convergent validity assessment based on the AVE value as the evaluation criterion revealed that three measures did not exhibit acceptable convergent validity. Based on item loadings we decided to delete one item of Scepticism towards advertising *(Advertisements are different from TV programs in the way they try to influence you),* two items of Ad avoidance *(Switch channels during commercials* and *Fast forward commercials)* and one item of Attitudes towards advertising *(Advertising should be more closely regulated)* to increase convergent validities of the measures. Composite reliabilities all exceeded the acceptance value of .80 (Höck & Ringle, 2006). The coefficient of determination (*R*^2^) was used to measure the overall size of the effect in the model. The value of *R*^2^ was 22.4% for Scepticism towards advertising, 8.7% for Ad avoidance, 3.9% for Compulsive buying and 1.7% for Attitudes towards advertising (see Table 5).

**Figure 2.**
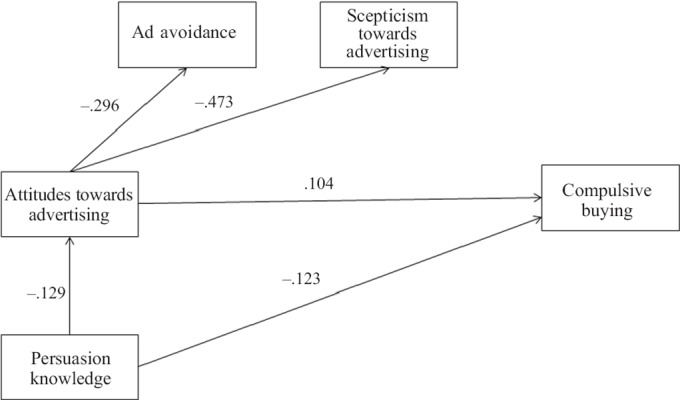
Advertising and compulsive buying – results of the SEM analysis

*Note:* Only significant paths are indicated in the graph.

The bootstrap samples were used to estimate the statistical significance of the PLS path model coefficients. The option of 5000 bootstrap samples was specified. Since the number of bootstrapping subsamples is large, the results approximate normality and we can use normal (Gaussian) quantiles to determine critical *t*-values for significance testing. According to the results five paths were significant: Attitudes towards advertising appeared to have the strongest relation with Scepticism towards advertising (b = –.479, *p* < .01), followed by Ad avoidance (b = –.445, *p* < .01) (H3 confirmed), and Compulsive buying (b = .112, *p* < .05) (H1 confirmed). Persuasion knowledge also appeared to be related to Attitudes towards advertising (b = –.168, *p* < .01) and Compulsive buying (b = –.172, *p* < .01). Hypotheses H2 and H4 were not confirmed by the study. The overview of the results is presented in Figure 2.

**Figure 3.**
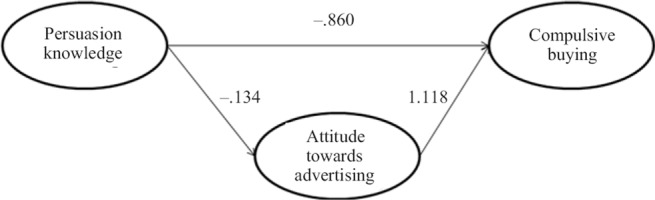
Persuasion knowledge and compulsive buying – the mediating role of attitudes towards advertising (results of the mediation analysis)

*Note:* Adjusted *R*^2^ for the current model was .028, *p* < .001. Mentioned in the graph are the b coefficients for the significant paths (*p* < .01).

The mediating effect of attitudes towards advertising specified in Hypothesis 5 was tested by employing the tests of mediation suggested by Zhao, Lynch and Chen (2010) and Hayes and Preacher (2011). Based on this framework, three separate regressions were estimated: 1) the effect of persuasion knowledge on attitudes towards advertising; 2) the effect of persuasion knowledge on compulsive buying and 3) the effect of attitudes towards advertising on compulsive buying. Figure 3 provides an overview of the mediation analysis.

The results of the analysis indicate that attitudes towards advertising mediates the relation between PK and CB (*CI_low_* – .301; *CI_up_* – .035).^1^ More in detail, persuasion knowledge has an indirect effect on CB through attitudes towards advertising (–.149, s.e. = .069), supporting Hypothesis 5. However, as also a direct relationship between PK and CB appeared to be significant (b = –.860, *p* = .008), we should talk about ‘competitive mediation’ (Zhao et al. 2010).

**Table 5.** Patch coefficients and *t*-values

	Compulsive buying	Attitudes towards advertising	Scepticism towards advertising	Ad avoidance
	β/t	β/t	β/t	β/t
Attitudes towards advertising	.112/1.956^*^		–.479/11.066^**^	–.445/7.361^**^
Scepticism towards advertising	.016/.273			
Ad avoidance	–.049/1.146			
Persuasion knowledge	–.172/2.570^*^	–.168/2.892^**^		

*Note:* ^*^ *p* < .05; ^**^ *p* < .01.

**Table 6.** The influence of advertising on compulsive and non-compulsive buyers

	Do you think you are exposed to advertising?				Do you think you are influence by advertising?			
	Non-compulsive		Compulsive		Non-compulsive		Compulsive	
	%	#	%	#	%	#	%	#
Never or seldom	4.3	22	2.1	1	18.4	95	4.2	2
Sometimes	12.8	66	14.6	7	56.3	291	43.8	21
Often	59.0	305	54.2	26	23.4	121	50.0	24
Always	24.0	124	29.2	14	1.9	10	2.1	1
Total	100.0	517	100.0	48	100.0	517	100.0	48
Chi-square	1.279 (*p* = .734)				18.526 (*p* < .001)			

**Table 7.** The influence of different advertising media on compulsive and non-compulsive buyers

	Via which media do you feel influenced by advertising?					
	Non-compulsive		Compulsive		Chi-square	*p*
	%	#	%	#		
TV	85.5	442	85.4	41	0.000	.989
Magazines	72.1	373	85.4	41	3.949	.047
Billboards	33.8	175	58.3	28	11.438	.001
Internet	51.8	268	72.9	35	7.848	.005

To investigate whether compulsive buyers feel more vulnerable to advertising than non-compulsive buyers, we divided the respondents in two separate groups – compulsive and non-compulsive buyers. According to Ridgway et al. (2008) respondents who obtained 25 or more points on the compulsive buying scale should be classified as compulsive buyers. In the current study 48 respondents (8.5% of the sample) could be considered compulsive buyers. Tables 6 and 7 present the results of the comparison between both groups as to how exposed they feel to advertising in different media and how influenced they feel by these different advertising channels.

The results show that the majority of the respondents feel to be often exposed to advertising. With this regard no differences between compulsive and non-compulsive buyers can be discerned (*p* = .734). On the other hand, compulsive buyers admit to feel more often influenced by advertising than non-compulsive buyers (*p* < .001). Specifically, more compulsive than non-compulsive buyers admit to be influenced by ads in magazines (85.4% vs. 72.1%; *p* = .047), billboards (58.3% vs. 33.8%; *p* = .001) and the Internet (72.9% vs. 51.8%; *p* = .005). No differences have been found with regard to TV ads (85.4% vs. 85.5%; *p* = .989).

## Discussion

First of all compulsive buying appears to be positively related to the attitudes towards advertising, supporting Hypothesis 1. Contrary to what we expected, scepticism toward advertising is not directly related to CB (H2 rejected). We also predicted a negative relation between the attitudes towards advertising and ad avoidance. This hypothesis can be confirmed by the analysis (H3 confirmed), meaning that the more negative the general attitudes towards advertising, the more chance to engage in ad avoidance. On the other hand, however, ad avoidance appeared to be unrelated to compulsive buying (H4 rejected). This is an interesting finding which means that avoiding ads does not prevent from engaging in CB.

Advertising is an important element of modern life: consumers are exposed to hundreds of commercial messages every day (Arens et al., 2007) via TV, magazines and newspapers, billboards and on the Internet. According to Shavitt et al. (1998) attitudes towards advertising can be inconsistent across different groups in the population. This appears to be true when looking at the results of the current study comparing compulsive with non-compulsive buyers. This paper provides evidence that positive attitudes towards advertising can lead to compulsive buying. An important factor in this relation is persuasion knowledge. The study results show (for a comprehensive overview see Table 8) that people lower in persuasion knowledge exhibit more positive attitudes towards advertising what can subsequently encourage them to engage in compulsive buying. High scores on scepticism towards advertising and ad avoidance among Belgian consumers in our sample, however, point to a need for advertisers to modify their practices in order to gain more consumer trust. This paper shows that advertising in particular attracts and seems to influence an already disadvantaged group of people – compulsive buyers. Especially magazine, billboard and Internet ads appear to seduce compulsive buyers more than their non-compulsive counterparts. It is therefore important to educate this underprivileged group of consumers about the influence that advertising might have on their maladaptive behaviour, which often leads to financial and also partnership problems. Gaining more persuasion knowledge could be a solution for compulsive buyers as knowing what marketing tactics are used in advertising may reduce their compulsive need to acquire the advertised products.

**Table 8.** Results overview

	Hypothesis	Test	Conclusion
Compulsive buying	*Pearson r*		
Attitudes towards advertising	H1: positive relation	.139^**^	Confirmed
Scepticism towards advertising	H2: negative relation	–.068	Not confirmed
Ad avoidance	H4: negative relation	–.058	Not confirmed
Attitudes towards advertising	*Pearson r*		
Ad avoidance	H3: negative relation	–.355^**^	Confirmed
Mediation through:		*Effect*	
Attitudes towards advertising	H5: PK * CB	–.149^*^	Confirmed
Influence of advertisement via:		*Chi-square*	
TV	H6a: CB > nonCB	.989	Not confirmed
Magazines	H6b: CB > nonCB	.047	Confirmed
Billboards	H6c: CB > nonCB	.001	Confirmed
Internet	H6d: CB > nonCB	.005	Confirmed

*Note:* ^*^ CIlow – .301, Clup – .035; ^**^ *p* < .01 (two-tailed).

In this era of social responsibility more attention has been given to ethics in marketing practices. There appears to be a difficult task to be fulfilled by managers, whose mission is no longer solely striving to obtain the best profits for their companies, but at the same time assuring that their practices are fair and benefit society. As the current research results show that an underprivileged group of consumers, namely compulsive buyers, are particularly vulnerable to and affected by advertising, it is recommended that more attention is paid to this problem. We encourage a thorough reflection on the way advertising messages could be adjusted in order to prevent compulsive buyers from spending more than they need or can afford.

## Limitations and Further Research

Although we believe that the current research extends our understanding of external triggers to compulsive buying, further investigation is encouraged. First of all, a convenience sample has been used in the study, what limits the generalizability of the study. Moreover, the research results of the current study are contrary to what has been found in a previous study by Kwak et al. (2002), who found a negative relation between attitudes towards advertising and compulsive buying. One of the reasons can be the advertising context under investigation. The focus of Kwak et al.’s (2002) research was on TV advertising, whereas our research examined advertising in a broader context, including magazines, billboard and Internet ads. This reasoning finds some support in our study results, which demonstrate that compulsive and non-compulsive buyers feel equally influenced by TV advertising but compulsive consumers seem to feel more vulnerable to ads on the Internet, billboards and magazines in comparison to their non-compulsive counterparts. Our investigation of the attitudes towards advertising in this broader context could therefore have resulted in these different findings.

Another possible reason for this inconsistency could be a cultural difference. Van der Wurff, Bakker and Picard (2008) for example showed that countries differ in their advertising intensity and newspaper share, and so can be grouped in 3 advertising cultures, namely high advertising intensity–low newspaper share; medium advertising intensity–high newspaper share and low advertising intensity–low newspaper share. An analysis of 21 countries, divided over those 3 different advertising cultures, indicated that in a country where newspapers have a larger share of the advertising budget, this budget is more strongly related to the economic situation as compared to countries where newspapers make out a lower share of the advertising budget. Moreover, the extent of advertising Belgian consumers is exposed to, is still much smaller than what American consumers have to bear. Research on this topic in other countries is therefore encouraged.

Our study results show that a lack of persuasion knowledge is an important factor in determining compulsive buying. In our mediation analysis the relationship between PK and CB was mediated by attitudes towards advertising. This confirms the theory of PK which says that PK leads to attitude formation and can subsequently influence behaviour. However, next to the mediating effect of attitudes towards advertising, PK still had a direct relation to CB which might point to the existence of other factors mediating this relationship. Further research should examine other possible mediators of this relationship.

Lastly, the advertising variables explained 3.9% of the variance in CB. This result is significant in consumer studies and indicates that the influence of advertising on CB should not be overlooked. However, since CB is mainly motivated internally, internal factors such as materialistic values or depression explain CB much better than the external factors. This has to be born in mind when interpreting the results of this study.

## Footnotes

^1^ In the Hayes and Preacher (2011) mediation analysis, confidence intervals (CI_low_ and CI_up_) instead of *p* values are used for significance testing of the effect. Number of samples used for indirect effect confidence intervals is 1000 and the level of confidence for confidence intervals 95.000 (95% confidence interval reflects a significance level of 0.05).
